# Factors influencing the resilience of smallholder livestock farmers to agricultural drought in South Africa: Implication for adaptive capabilities

**DOI:** 10.4102/jamba.v11i1.805

**Published:** 2019-10-23

**Authors:** Ringetani Maltou, Yonas T. Bahta

**Affiliations:** 1 Department of Agricultural Economics, Faculty of Natural and Agricultural Sciences, University of the Free State, Bloemfontein, South Africa

**Keywords:** resilience, agricultural drought, smallholder livestock farmers, agricultural drought resilience index

## Abstract

The aim of this study was to determine the factors that influence the resilience of smallholder farming households in the Northern Cape province of South Africa. This study utilised primary data collected from 207 smallholder livestock farmers and the probit model. The study contributes to the existing literature by constructing an agricultural drought resilience index (ADRI) as an outcome variable in order to determine the factors that influence the resilience of smallholder farmers to agricultural drought. The result revealed that only 9% of the smallholder livestock farmers were resilient to agricultural drought. Farming households with access to credit; farmers who received assistance from the government (such as training and feed) during drought and farmers who are part of a co-operative proved to be more resilient to agricultural drought. The policy implication of the findings of this study is for government and key role players in the industry to target disadvantaged smallholder farmers to build their resilience by enhancing their persistence and adaptability. Some of the assistance could be in the form of supplying fodder, strengthening access to agricultural credit and farm input, enhancing smallholder farmers’ involvement in agricultural drought resilience activities by giving training and disseminating information. Finally, yet importantly, there is a need to enhance the resilience of farmers by educating farmers about the importance of getting involved in cooperatives and social networks. Furthermore, the findings of this study will help policymakers and stakeholders to formulate strategies and policy interventions that will boost smallholder farmers’ resilience to agricultural drought.

## Introduction

Livestock production is an important agricultural commodity for food security providing the world with 17% kilocalorie consumption and 33% protein consumption contributing to the livelihoods of 1.0 billion poor people globally (Rojas-Downing et al. 2017). In South Africa, the production of livestock has great potential to alleviate food insecurity and poverty (Mapiliyao et al. 2012). The livestock industry contributes to approximately 48% of South Africa’s agricultural output and employs approximately 500 000 people nationwide (Department of Agriculture, Forestry and Fisheries [DAFF] 2016).

Livestock is by far the largest sub-sector in the South African agricultural sector, contributing an estimated 25% – 30% of the total agricultural output per year. Cattle, sheep and goat farming occupy approximately 53% of all agricultural land in South Africa (Blignaut et al. 2014). Around 33.8 million hectares in the Northern Cape are classified as farmland, with about 86% of the land used for grazing livestock (Klynveld Peat Marwick Goerdeler [KPMG] 2012). The province has more focus on livestock farming when compared to the other agricultural activities. Sheep and cattle production plays a very important role in the South African livestock industry because it is a source of income in cash and therefore contributes to farmers’ livelihood.

However, as with the rest of the African region, the agricultural sector in South Africa is vulnerable to drought (FAO 2013). Prolonged droughts are regular and recurrent features affecting smallholder and emerging farmers and are one of the most important disasters in economic, social and environmental terms in Southern Africa including South Africa (Buckland, Eele & Mugwara 2000; Rouault & Richard 2003). Recurrent drought is a challenge for smallholder farmers because of unavailability of resources. Smallholder farmers in South Africa are faced with constraints that have undermined their potential to produce adequate output. Some of the notable constraints include higher demand for agricultural land, lack of capital, rising prices of farm inputs, low prices of farm output which, together with other challenges such as lack of assets, information, access to services, poor physical and institutional infrastructure, have resulted in a cost-price squeeze for farmers (DAFF 2012).

The 2015/2016 drought resulting from very strong El Niño conditions was comparable with the droughts of 1933 and 1982. According to the Department of Water and Sanitation (DWS 2015), approximately 173 out of 1628 water supply schemes across the country were affected by drought. These water schemes supply approximately 2.7 million households in South Africa. Drought in South Africa costed farmers’ losses up to R10 million in 2015 (Bahta, Jordaan & Muyambo 2016). Agricultural production declined by 8.4% during the year 2015. The decline in agricultural production was attributed to the worst drought conditions which intensified in January 2015. The livestock industry (cattle and sheep) was one of the industries that were severely affected by drought, with a reduction of 15% in the national herd stock (Agri SA 2016). The intensity of drought creates additional stress on livestock smallholder farmers’ cash flow, mental status and their resilience.^1^

Agricultural systems are complex social ecological systems that are substantially affected and increasingly threatened by various hazards, for example, climate change (Boko et al. 2007), hence are often assessed with respect to their resilience and/or adaptability (Callo-Concha & Ewert 2014). With growing concerns about climate and environmental changes, and increase in social, economic or political upheavals, the concept of resilience has become popular across a range of sectors as a way to understand and respond to our surprise-riddled world (Reyers & Moore 2017).

Resilience is linked to dynamics of social systems such as adaptability, transformability, capacity of communities and complex socio-ecological systems to learn, to cope and adapt and transform in the face of shocks and stresses. Resilience would be defined as the capacity to bounce back. Resilience emphasises the interplay between gradual change and abrupt change. It brings attention to the capacity to live with change in turbulent times (Colburn & Seara 2011; Folke 2017). Resilience is a property of complex adaptive systems. It is the whole system that enables or constrains its ability to continue to develop with change; it is about increasing the capacity to continue on a particular development path in the face of change that is both incremental and abrupt, expected and surprising. It is about adapting, improving and innovating on that path (Reyers & Moore 2017).

Adaptive capacity (capacity to adapt, capacity of response or coping capacity) refers to the ability to become adapted, to cope with the constraints that a system faces or, as in the context of climate change, it is the ‘adjustment in natural or human systems to actual or expected climate stimuli and their effects’ (IPCC 2007). Smit and Wandel (2006) affirm that adaptation refers to a process, action or outcome in a system in order to better cope with, manage or adjust to some changing condition, hazard, risk or opportunity.

Existing international and national studies, such as those of Vetter (2009); Sallu, Twyman and Stringer (2010); Banda et al. (2016); Mdungela, Bahta and Jordaan (2017), and Jiri, Mafongoya and Chivenge (2017) focused on the application and relevance of resilience; understanding and managing ecosystem change and enhancing the capacity of land users to adapt to droughts; identifying factors that influence resilience to drought among smallholder crop farmers; assessing livelihood dynamics and factors influencing farmers’ choice of coping strategies. None of them determines the factors that influence the resilience of smallholder livestock farming households to agricultural drought using the agricultural drought resilience index (ADRI) as the dependent variable. The current knowledge regarding ADRI is not enough. Therefore, the present study attempts to fill this gap in knowledge and literature.

The main objective of the study was to determine the factors that influence the resilience of smallholder^2^ farming households to agricultural drought in Northern Cape province of South Africa. The findings of this study will help policymakers to formulate appropriate policy interventions to sustain smallholder livestock farmers against the exposures of drought, which is a threat to livelihood, food security, survival and achieving the sustainable development goal (SDG) of ending hunger and poverty by 2030. The research which is reported in this article is part of a more comprehensive research project on ‘Household resilience to agricultural drought in the Northern Cape province of South Africa’ Contract Number/Project Number (TTK170510230380).

## Methodology

### Study area

The study was conducted in the Frances Baard District Municipality which is the smallest district located in the eastern portion of the Northern Cape province of South Africa. The district accounts for only 3.4% of its geographical area but accommodates the largest proportion of the province’s population with 3085 people per square km. Frances Baard District Municipality comprises four local municipalities which are Dikgatlong (2377.6 km²), Magareng (1541.6 km²), Phokwane (833.9 km²) and Sol Plaatje (1877.1 km²). Setswana, Afrikaans, English and IsiXhosa are the dominant languages in the district. Approximately 48 300 households were engaged in agriculture during 2016 in the Northern Cape province (Statistics South Africa [Stats SA] 2016). A large number of these households were engaged in animal farming (74.9%), while other households were engaged in cultivating only crops (15%) and mixed farming (10%). Households in the Northern Cape province practice agriculture in their backyards, farmland and communal land.

### Sampling procedure and data description

A multiple-stage sampling technique was employed. First, the Northern Cape province was chosen from the nine provinces of South Africa because they represented the main livestock-producing provinces. Furthermore, the province has the potential to produce adequate livestock as compared to crops. The Northern Cape province was also chosen because they had been declared a disaster zone by the South African government in the 2017/2018 calendar year.

In the second stage of the sampling procedure, four district municipalities from the province, namely, Dikgatlong; Magareng; Sol Plaatjie and Phokwane were chosen randomly. Smallholder livestock farmers were selected from the Northern Cape Department of Agriculture, Forestry and Fisheries (2018). They received the assistance from the government because of severe drought in the calendar year 2015–2016 (Table 1).

**TABLE 1:** Number of farmers who received assistance from the government and sampling procedure.

Local municipality	Number of farmers	Share of farmers (number of farmers/total)%	Number of sample (percentage *total sample size [207])
Dikgatlong	347	40	83
Magareng	119	14	29
Sol Plaatjie	263	30	62
Phokwane	139	16	33

**Total**	**868**	**-**	**207**

*Source*: Adapted from Northern Cape Department of Agriculture, Forestry and Fisheries (NDAFF), 2018, *Beneficiaries of drought relief program*, Northern Cape Department of Agriculture, Forestry and Fisheries, Northern Cape, Kimberly

Note: The asterisk (*) represents multiplication.

To come up with a correct number of sample representation a simple random sampling formula was applied for a finite population. To calculate appropriate sample sizes for a survey, for continuous and categorical data, formulae were developed by Cochran (1977). The questionnaire that was used, collected both continuous and categorical data; thus, to ensure that the sample size is appropriate, the calculation for categorical data will be used to calculate the sample size (Bartlett, Kotrlik & Higgins 2001) and is expressed as Equation 1.

Total sample size is calculated using:

$$M_{o}=\frac{u^{2}*fi}{e^{2}}$$ [Eqn 1]

where: M_*0*_ = sample size

*u* = the level of risk the researcher is willing to take (margin of error may exceed the acceptable margin of error) – for the selected alpha level

*(f)(i)* = estimate of variance = 0.25 (maximum possible proportion [0.5]*1-maximum possible proportion [0.5] produces maximum possible sample size)

*e* = acceptable margin of error for proportion being estimated = 0.05

Alpha level (*u*) of 1.65-estimated variance of 0.5 and an error level of 0.05 were used; the formula would look as follows:

$$M_{o}=\frac{{\left(1.65\right)}^{2}*(0.5)(0.5)}{{\left(0.05\right)}^{2}}=272$$ [Eqn 2]

This results in a sample size of 272 respondents (indicating that the sample size exceeds 5% of the population); hence, the correctional formula (Equation 3) of Cochran (1977) was applied to calculate the final sample size:

$$\begin{array}{l} M_{1}=\frac{M_{0}}{1+\frac{M_{0}}{\text{population}}}\\ M_{1}=\frac{272}{1+\frac{272}{868}}=207 \end{array}$$ [Eqn 3]

where *M*_o_ is the sample size, *M*_1_ is the final sample size. Based on the above formula, 207 smallholder livestock farmers were selected from the Northern Cape province of South Africa for a face-to-face interview from July to September 2018 using a structured questionnaire including questions related to access and availability of insurance.

### Data analysis and method

After data was collected, it was captured on excel. Statistical Package for the Social Science (SPSS) and stata packages were used for all statistical analyses. To calculate the outcome variables (ADRI), a principal components analysis (PCA) was applied. Principal components analysis is used to aggregate four production and consumption related indicators into the ADRI. Principal components analysis is a method applied to reduce a large set of variables to smaller variables by taking into consideration the variance of original data or variables (Beaumont 2012; Holland 2008). The analysis was done using the SPSS software.

The proposed variables are livestock production produced by smallholder farmers in a normal year without agricultural drought (LVPNYWOAD), livestock produced with agricultural drought (a bad year) (LVPWAD), the number of months a household consumes food produced by the household in a normal year (without agricultural drought) (NMHCFNWOAD) and the number of months a household consumes food produced by the household in a bad year (with agricultural drought) (NMHCFWAD). Principal components analysis was utilised to aggregate these four variables.

The four indicators (LVPNYWOAD, LVPWAD, NMHCFNWOAD and NMHCFWAD) will aggregate into ADRI using Equation 4 and description of variables in the formula depicted in Table 2:

$$\text{ADRI}=W_{\text{n}}P_{\text{n}}+W_{\text{d}}P_{\text{d}}+W_{\text{cn}}M_{\text{n}}+W_{\text{cd}}M_{\text{d}}$$ [Eqn 4]

**TABLE 2:** Description variables as dependent and independent in Equation 4.

Variables	Description
**Dependant variable**	
ADRI	Denotes the agricultural drought resilience index
**Explanatory variables**	
*W*	Represents weights derived from the component loadings from the first principal components. The data from which the components will be derived to have a zero mean and unit variance
*W*_n_*P*_n_	Denotes the weight for livestock production in a normal year (without agricultural drought) multiplied by the actual amount of livestock production produced in good year (without agricultural drought)
*W*_d_*P*_d_	Denotes the weight for livestock production in a drought year (with agricultural drought) multiplied by the actual amount of livestock production produced in drought year (with agricultural drought)
*W*_cn_*M*	Denoted the weight for the number of months a household remains with household-produced food multiplied by the number of months the household consumes household-produced food in a normal year (without agricultural drought)
*W*_cd_*M*_d_	Represents the weight for the number of months a household remains with household**-**produced food during a drought year multiplied by the actual number of months a household remains with household-produced food in a drought year

ADRI, agricultural drought resilience index.

All variables are expected to correlate positively with drought resilience. This is because an increase in any one of the variables was expected to be associated with an improvement in the well-being of the farming household.

A probit regression procedure was used to identify the factors that determine the resilience of households to agricultural drought in the Northern Cape province. This method has been used by several authors to study determinants of household resilience to dry spells and drought in Malawi (Banda et al. 2016). According to Walsh-Dilley, Wolford and McCarthy (2013), the resilience framework focuses on understanding and promoting the capacity of local communities to respond, negotiate and transform shocks such that disturbances do not initiate a downward spiral and may even provide opportunities for improvement. The probit model is expressed as:

$$Y_{i}=\alpha_{0}+\alpha_{1}X_{1}+\alpha_{2}X_{2}\;\ldots \;\ldots \;\ldots \;+\mu_{i}$$ [Eqn 5]

where *Y*_*i*_ is the dependent variable (outcome variable), *α* is the parameter to be estimated, *X* is the independent variable and *µ*_*i*_ is the error term. More specifically, the model expressed in detailed as:

$$\begin{array}{l} R_{i}=\alpha_{0}+\alpha_{1}\text{Age}+\alpha_{2}\text{Gender}+\alpha_{3}M\text{Status}+\alpha_{4}\text{Funding}+\\ \alpha_{5}\text{Relatives}+\alpha_{6}\text{Institution}L+\alpha_{7}c{\text{COMMUNITY}}_{c}\\ \text{OLABORATION}+\alpha_{8}g\text{VMENT}+\alpha_{9}T\text{source}+\alpha_{10}\text{NASS}+\\ \alpha_{11}N\text{live}+\alpha_{12}O\text{assets}+\alpha_{13}\text{COOP}+\alpha_{14}C\text{strategy}+\\ \alpha_{15}A\text{Strategy}+\alpha_{16}\text{ESUPP}+\alpha_{17}\text{GOVI}+\alpha_{18}\text{FEXP}+\\ \alpha_{19}\text{EDUC}+\alpha_{20}\text{DR}+\alpha_{21}\text{Sus}+\alpha_{22}\text{HM}+\alpha_{24}\text{RSUPP}+\mu_{i} \end{array}$$ [Eqn 6]

The description of each outcome and independent variable is illustrated in Table 3.

**TABLE 3:** Description of variables used in the probit model expected sign.

Variables	Description	Expected sign
**Dependant variable**		
ADRI	1 if Yes, 0 if No	-
**Explanatory variables**		
Age	Number of years	+
Gender	0 if female and 1 if male	+
Marital status (Mstatus)	1 if single, 2 if married, 3 if a widow, 4 if divorced, 5 if separated, 6 if other	+
Education (EDUC)	Number of years	+
Farming experience (FEXP)	Number of years	+
Funding	Family savings = 1, borrowings = 2 and other = 3	+/-
Household members (HM)	Number of members	+
Relatives	0 if No, 1 if Yes	+/-
Relative support (RSUPP)	Amount in Rands	+
Other water sources (Tsource)	0 if No, 1 if Yes	+/-
Drought response (DR)	Sell livestock = 1, Buy more livestock = 2, Stop production = 3, Sell assets = 4, Other = 5	+
Number of livestock (Nlive)	Number of livestock	+/-
Other assets (Oassets)	0 if No, 1 if Yes	+/-
Co-operative (COOP)	0 if No, 1 if Yes	+
Coping strategies (CStrategy)	Migrate = 1, ask for food = 2, sell livestock = 3, look for a job = 4, lessee part of the farm = 5, other = 6	+
Adaptive strategies (AStrategy)	Search for breeds that resist drought = 1, diversify farm activities = 2, livelihood diversification = 3, adopt conservation agriculture = 4, other (specify) = 5	+
Neighbour assistance (NASS)	0 if No, 1 if Yes	+/-
Enough support (ESUPP)	0 if No, 1 if Yes	+/-
Institutions help (InstitutionL-Credit)	0 if No, 1 if Yes	+
Community collaboration (cCOMMUNITYcOLABORATION)	0 if No, 1 if Yes	+/-
Government assistance (gVMENT)	0 if No, 1 if Yes	+
Government Interest (GOVI)	0 if No, 1 if Yes	+
Sustaining natural resource (Sus)	0 if No, 1 if Yes	+

ADRI, agricultural drought resilience index.

### Ethical considerations

This article followed all ethical standards for research without direct contact with human or animal subjects.

## Results and discussion

### Estimation of agricultural drought resilience index

As indicated in Table 4, an average household resilience index in the Frances Baard District Municipality was -6.31; this result implies that the average households in the Frances Baard District Municipality are not resilient to agricultural drought. Furthermore, the result confirms that only 18 smallholder livestock farmers (accounting for 9%) of the farming households were resilient to agricultural drought. The remaining 189 smallholder livestock farmers (accounting 91%) were not resilient to agricultural drought. This implies that the farmers need assistance such as funding, fodder and other farm inputs from the government during the dry periods; farmers also need an assistance from neighbours – by allowing access to water or letting livestock of the other farmer to graze together and, last but not the least, farmers also need an assistance from the community in the form of resource exchange and effective communication among community members (farmers), and that forms part of components of community resilience. From the sample, 72% of farmers who were resilient to agricultural drought were males, whereas 28% were females. On the other hand, 82% of the non-resilient farmers were males, and 18% were females. This implies that women do not have the necessary resources as compared to male farmers to enhance their resilience. A significant difference was noted between the educational level of the farming household heads; 78% of the farmers who are resilient to agricultural drought were educated (received formal education), whereas 22% of the farmers did not have any formal education. This implies that education helps to gather the necessary information related to drought and drought strategies in order to plan to enhance resilience.

**TABLE 4:** Summary statistics for the agricultural drought resilience index for the Frances Baard District Municipality.

Variable	*N*	Mean	Standard deviation	Minimum	Maximum
ADRI	207	−6.31	6.90	−2.43	6.69
ADRI > 0	18	0.51	1.87	0.14	6.69
ADRI < 0	189	−7.00	6.88	−2.43	−0.008

ADRI, agricultural drought resilience index.

### Enhancing resilience of smallholder livestock farmers to agricultural drought

Table 5 summarises the key factors that affect the farming household’s resilience to agricultural drought.

**TABLE 5:** Factors that affect the farming household’s resilience to agricultural drought.

ADRI	Coefficient	Standard error	*p*	Margin effect	*p*
Age	0.001	0.015	0.529	0.001	0.529
Gender	−0.731	0.428	0.088*	−0.084	0.083*
Mstatus	0.123	0.3	0.682	0.014	0.682
Funding	0.088	0.198	0.658	0.01	0.658
Relatives	−0.301	0.396	0.447	−0.035	0.444
Institution (credit)	1.038	0.498	0.037**	0.119	0.033**
cCOMMUNITYcOLABORATION	−0.442	0.424	0.298	−0.051	0.296
gVMENT	−1.341	0.794	0.091*	−0.154	0.088*
Tsource	0.145	0.199	0.467	0.017	0.467
NASS	−1.482	0.969	0.126	−0.17	0.126
Nlive	0.001	0.002	0.553	0	0.553
Oassets	−0.438	0.417	0.293	−0.05	0.292
COOP	−0.886	0.509	0.082*	−0.102	0.076*
Cstrategy	−0.143	0.127	0.26	0.016	0.259
AStrategy	0.021	0.122	0.808	−0.003	0.808
ESUPP	−0.155	0.472	0.742	0. 018	0.742
GOVI	1.111	0.784	0.156	0.128	0.152
FEXP	0.002	0.023	0.932	0	0.932
EDUC	−0.365	0.204	0.073*	−0.042	0.067*
DR	−0.042	0.104	0.686	−0.005	0.686
Sus	−0.543	0.494	0.272	−0.062	0.267
HM	−0.078	0.068	0.251	−0.009	0.247
RSUPP	0.774	0.439	0.078*	0.089	0.073*
Constant	1.823	1.821	0.317	-	-

Note: *p*-value – the last *p*-value indicated after the calculation of marginal effects.

ADRI, agricultural drought resilience index; EDUC, education; FEXP, farming experience; HM, household members; RSUPP, relative support; Tsource, other water sources; Nlive, number of livestock; Oassets, other assets; COOP, co-operative; CStrategy, coping strategies; AStrategy, adaptive strategies; NASS, neighbour assistance; ESUPP, enough support; gVMENT, government assistance; GOVI, government interest; Sus, Sustaining natural resource; cCOMMUNITYcOLABORATION, Community collaboration; DR, drought response; AStrategy, adaptive strategies.

*, ** , Significant at the 10% and 5% level.

The gender of the farming household head was found to have a negative significant influence on the resilience of a household to agricultural drought. Banda et al. (2016) also found that there is a negative correlation between gender and resilience, but at an insignificant level. These results suggest farming households headed by males were more likely to be less vulnerable to agricultural drought when compared to farming households headed by females. However, Andersen and Cardona (2014) and Jiri et al. (2017) concluded that gender had an insignificant effect on the resilience of a farming household to agricultural drought.

The finding on the influence of education on enhancing the resilience of a farming household has been mixed with some studies showing no influence and others showing positive or negative influence. This study found that educated farmers are unlikely to enhance their resilience to agricultural drought better than the non-educated farmers. This is similar to the findings of Andersen and Cardona (2014) who indicated that education has a very small, barely significant effect on resilience. An increase in the respondents’ years of formal education by 1 results in 0.365 reductions of the farming household resilience. This is also in line with the findings of Banda et al. (2016) and Jiri et al. (2017) who find that the education level of the household head had no significant influence on the adaptation to climate change.

The farming household received financial assistance from immediate family members living outside the households that tend to be resilient compared to households that do not have any financial assistance from immediate family members living outside the households. These findings were consistent with findings by Andersen and Cardona (2014) and Banda et al. (2016) who found that households that receive financial support from relatives tend to be more resilient compared to those not receiving any financial support. The assistance has an implication on household purchasing and consumption of food when drought strikes. The marginal effect coefficient of 0.089 implies that an increase in the amount (in Rands) received from immediate family members living outside the household results in a corresponding increase in the probability of a household enhancing its resilience by 0.087. The more the farming household receives financial support from relatives the more are its chance of being resilient to agricultural drought.

Institutions have a positive impact on enhancing resilience to agricultural drought. Jiri et al. (2017) highlighted that; access to credit (institution) positively influenced household resilience to agricultural drought. Access to credit would allow farmers to purchase enough inputs such as feeds and medicines for their livestock. These results suggest that farming households with access to any form of credit or assistance from other institutions such as agricultural private organisations or banks are more likely to enhance their resilience to agricultural drought when compared to those farming households that do not have access to credit or any other form of credit.

The co-operative variable has a negative effect on the resilience of a farming household to agricultural drought and it is significant. This implies that respondents that are part of a co-operative are most likely to be resilient to agricultural drought. Farmers that are part of a co-operative are able to assist each other (allowing livestock to graze on each other’s farms and if the other farmer does not have access to water, they can share), share information or knowledge and are able to buy feed in bulk. This was consistent with the findings of Keil et al. (2008) who found that households involved in a number of village organisations positively influences their resilience.

Government assistance was also found to be significant and negatively related to households resilient to agricultural drought. These results show that farming households that receive assistance from the government during dry periods are very likely to be resilient. According to the farming households that were interviewed, during the 2015/2016 drought, they received assistance from the government (coupons to purchase feed) depending on the number of livestock the farmer has. However, farmers argued that this was not enough as agricultural drought lasts for a longer period of time and although the government assisted it was very late; some farmers had already started losing or selling livestock.

Government assistance can also include informing communities about weather predictions (e.g. the 2015/2016 drought was announced before it occurred), provide livestock management training during agricultural drought periods and regular farm visits by extension officials. Jiri et al. (2017) argued that access to extension information significantly affects the farmers’ decision to adapt to climate change. This means that farming households with government assistance (extension officials) are expected to be more resilient to agricultural drought because of better livestock management during this period.

Other variables that were employed in the model were insignificant including age, marital status, funding, community collaboration, other water sources, neighbour assistance, number of livestock, other assets, coping strategies, prior knowledge, enough support, farming experience, drought response, sustaining natural resources and number of household members. This implies that the influence of these variables (insignificant variables) on the dependent variable (ADRI) was less or had no influence compared to the influence of significant variables to dependent variables.

Besides, the above variables influences household resilience. Insurance is one of the main tools to enhance agricultural drought resilience. However, most livestock households indicated that they do not have insurance because it is expensive to insure livestock and they do not have enough resource to ensure that.

## Conclusion and recommendations

The results of this study have shown that government and policymakers should intervene by assisting farming households from Frances Baard District Municipality to enhance their resilience to agricultural drought. Only 9% of the livestock farming households from Frances Baard District Municipality were resilient to agricultural drought, while 91% of the farming households were not resilient. These results showed that for farmers to enhance their resilience to agricultural drought, the government should assist in terms of providing feeding, training (livestock management during dry periods), access to water and credit. To manage agricultural drought effectively farmers should have easy access to credit, enough land for grazing (enough camps), provide regular training (agricultural drought training) and assistance from the government should be provided on time before farmers become vulnerable.

Insurance is very important to any business; however, most livestock farmers have indicated that they do not have insurance because it is expensive to insure livestock. Policymakers should try considering developing an agricultural drought insurance, which is specifically for drought periods. A minimum premium should be set as agricultural drought occurs after a while, so that smallholder farmers can afford it.

## Footnotes

1 Resilience – refers to the capacity to absorb, predict, accommodate or recover from the effects of natural hazards in an efficient way through restoration, improvement or preservation, of its crucial basic structures and functions through risk management (IPCC 2012).

2 Smallholder farmers are farmers who grow subsistence livestock, completely relying on family labour and have limited resource endowment as compared to commercial farmers (DAFF 2012).
